# The association of medical mistrust, clinical trial knowledge, and perceived clinical trial risk with willingness to participate in health research among historically marginalized individuals living in New York City

**DOI:** 10.21203/rs.3.rs-6699898/v1

**Published:** 2025-06-30

**Authors:** Isabel Inez Curro, Laura C. Wyatt, Victoria Foster, Yousra Yusuf, Sonia Sifuentes, Perla Chebli, Julie A. Kranick, Simona C. Kwon, Chau Trinh-Shevrin, Madison N. LeCroy

**Affiliations:** City University of New York; NYU Grossman School of Medicine; NYU Grossman School of Medicine; NYU Grossman School of Medicine; NYU Grossman School of Medicine; NYU Grossman School of Medicine; NYU Grossman School of Medicine; NYU Grossman School of Medicine; NYU Grossman School of Medicine; NYU Grossman School of Medicine

**Keywords:** research participation, medical mistrust, health research knowledge, perceived research risk, racial and ethnic groups, health disparities

## Abstract

Medical mistrust, clinical trial knowledge, and clinical trial risk impact research participation, yet are rarely studied among racial and ethnic groups.

Data were from a cross-sectional survey (n = 1,788). Multinomial logistic regression models examined associations of medical mistrust, clinical trial knowledge, and clinical trial risk with willingness to participate in health research (Yes, No, Unsure) among Chinese, Korean, South Asian, Haitian, North American Latiné, South American Latiné, and Southwest Asian and North African (SWANA) NYC residents with one model per group

Overall, 46.1% of participants reported willingness to participate, ranging from 35.8% (Chinese participants) to 58.7% (South Asian participants). Increased mistrust was associated with less willingness among Chinese (OR: 1.06, 95%CI: 1.00, 1.12) and South American Latiné (OR: 1.15, 95%CI: 1.01, 1.30) participants; more willingness among Haitian participants (OR: 0.87, 95%CI: 0.81, 0.94); more uncertainty among Korean (OR: 1.13, 95%CI: 1.05, 1.22), South Asian (OR: 1.07 95%CI: 1.01, 1.12), and North American

Latiné (OR: 1.18, 95%CI: 1.09, 1.27) participants; and less uncertainty among Haitian (OR: 0.91, 95%CI: 0.84, 0.99) and SWANA (OR: 0.91, 95%CI:0.86, 0.97) participants. Knowledge was associated with more willingness for Haitian participants (OR: 2.77, 95%CI: 1.15, 6.65), less willingness for Chinese participants (OR: 0.55, 95%CI: 0.34, 0.88), and more uncertainty among South Asian (OR: 2.09, 95%CI: 1.07, 4.07) and SWANA (OR: 2.71, 95%CI: 1.21, 6.03) participants. Some risk and more willingness were linked for South American Latiné participants (OR: 0.14, 95%CI: 0.02, 0.85).

Associations varied by group. Studying diverse groups advances equitable research representation.

## Introduction

### Lack of Diverse Participation in Medical Research

The 1993 National Institutes of Health Revitalization Act requires women and ethnically and racially diverse individuals to be included in health research, aiming to reduce health disparities.^1^ Yet, from 1993–2018, 96% of U.S. randomized controlled trial (RCT) participants were White.^2^ Black/African American, Latiné (i.e., Spanish-speakers, those descended from Spanish-speakers, and those from or descended from Latin America), and Asian individuals were underrepresented in RCT’s by ≥ 450% compared to their respective share of the U.S. population (Black/African American: 2.2% vs. 12.5%; Latiné: 1.2% vs. 18.3%; Asian: 1.0% vs. 5.7%). According to the National Academies, this underrepresentation worsens health disparities, limits access to effective treatments, and could cost hundreds of billions of dollars in life expectancy, disability-free years, and labor force participation over the next three decades.^3^

### Medical Mistrust, Health Research Knowledge, and Perceived Health Research Risk

The U.S. medical system’s historical mistreatment of racial and ethnic groups contributes to ongoing health inequities.^4^ Egregious abuses—such as experimenting on enslaved Black bodies, the Tuskegee Syphilis Study, and forced sterilizations of Native, Latiné, and Black individuals—have fueled medical mistrust: the belief that medical institutions may act against patients’ best interests.^5^ Mistrust of both the U.S. healthcare and medical research institutions is common in the U.S.^5, 6^

Experiencing systemic racism and interacting with biased institutions are “basic causes” of medical mistrust.^5^ In the U.S. medical system, biases may be experienced as discrimination by one’s healthcare provider or inadequate culturally and linguistically appropriate health communication.^5, 7^ Mistrust hinders medical research participation, especially among racially and ethnically diverse U.S. residents.^6, 8, 9^

Health research knowledge and perceived risk associated with participation are also linked to participation willingness. George et al.’s 2014 systematic review on factors impacting research participation found that, besides medical mistrust, lack of health research knowledge was the only barrier shared across all included groups—African American, Latiné, Asian American, and Pacific Islander^4^—highlighting both the importance of knowledge to research participation and the heterogeneity of barriers to participation across differing groups. Among African American, Latiné, Asian American participants, fear of adverse effects was another barrier to participation, and perceived low risk of research participation was a facilitator.^4^

The impact of medical mistrust, health research knowledge, and perceived participation risk on participation willingness have been explored among some racial and ethnic U.S. groups, including African American and Latiné. However, few studies have examined these themes among further disaggregated ethnic, national, or regional subgroups (e.g., Chinese, Mexican). Further, other diverse groups residing in the U.S., including Korean, South Asian, Haitian, Central and South American, and Southwest Asian and North African (SWANA), are missing from the literature altogether.

### Data Disaggregation

Previous studies among Asian Americans have disaggregated data by racial and ethnic groups to reveal differences within broader aggregated groups.^10–13^ This study follows that approach by disaggregating data among a Black American subgroup: Haitian; Asian American subgroups: Chinese, Korean, and South Asian; and Latiné subgroups: North American Latiné (Latiné individuals identifying as from or as descendants of individuals from countries in North America, including Mexico, Central America, and the Caribbean) and South American Latiné (Latiné individuals identifying as from or as descendants of individuals from countries in South America).

### Latiné Migration

Latiné communities have a long history in New York City (NYC). Puerto Rican and Dominican communities comprised much of the first waves of Latiné immigration to NYC,^14^ sharing many of the same reasons for immigrating, including economic hardships, lack of employment opportunities, political factors, and family reunification.^15–17^ Mexican individuals followed soon after, and these three groups are the three most populus Latiné groups in NYC.^18^

Later, in the mid-1980’s, large waves of immigration from Central America to the U.S. began.^19^ Since then, the Central American immigrant population in the U.S. has grown tenfold, with individuals from El Salvador, Guatemala, and Honduras making up the majority of Central American migrating communities.^20^ NYC is home to the second highest concentration of Central American immigrants across all metropolitan areas in the U.S.^20^

Individuals immigrating to the U.S. from Mexico and Central America are often grouped together as Mexico has historically been the main entry point into the U.S. for these groups.^21^ Further, individuals from these countries experience similar causes for migration, including a dearth of job opportunities, poverty, and social and gender-based violence.^21^

NYC has the largest South American immigrant population of all U.S. cities.^22^ The Cold War era saw the first large waves of immigration into the U.S. from South American countries due to armed conflict and political and economic instability.^22^ Despite the overall growth of the South American Latiné immigrant population, there has been a steady decline in South American immigration over the past 20 years.^22^ This trend could be due to increased intracontinental immigration within South America and intercontinental immigration from South America to Spain.^21^

### Present Study

Using data from a community needs assessment designed to understand health-related challenges, needs, and resources of diverse and minoritized NYC populations, we examined whether medical mistrust or perceived research participation risk were associated with decreased willingness to participate in future health research and if health research knowledge was associated with increased willingness to participate among Chinese, Korean, South Asian, North American Latiné, South American Latiné, and SWANA NYC residents.

## Methods

This study used data collected via the Cancer Community Health Resources and Needs Assessment (Cancer CHRNA), a cross-sectional survey developed by NYU Langone Health Department of Population Health, NYU Langone Perlmutter Cancer Center, and 23 community partners. The survey was administered during October 2021-December 2022. Participants included 2,636 NYC residents aged 18 and older who were fluent in one of the ten survey languages (Arabic, Bangla, Chinese [Simplified or Traditional], English, Haitian Creole, Korean, Russian, Spanish, or Urdu). Cancer CHRNA was conducted in partnership with community-based organizations (CBOs) and used a multi-pronged convenience sampling approach. Asian Americans were intentionally oversampled to enable disaggregated data collection across ethnic subgroups. The institutional IRB approved the study protocol, survey, and recruitment materials, and participants were consented before participation. Data was obtained from NYU Langone Health via a data use agreement.

### Measures Outcome

*Willingness to participate in health research* was measured with the question “Would you ever participate in a health research study?” with answer choices Yes, No, Don’t know/Not sure, and Decline to state.

#### Exposures

*Medical mistrust* was examined as a continuous variable and was measured via responses on a 5-point Likert scale (1 = Strongly Disagree, 5 = Strongly Agree) to nine statements (e.g., “The Health Care System does its best to make patients’ health better”) sourced from the Health Care System Distrust Scale^23^ (see Appendix Table 1). Responses to statements (a), (c), (f), and (g) were reverse coded such that higher scores indicate more mistrust. When responses to two or less statements were missing, the mean of the answered statements was imputed for the missing statements. The nine responses were summed, creating a continuous variable ranging from 9 (least mistrust) to 45 (most mistrust). *Clinical trial* knowledge was measured with the question “How knowledgeable would you say you are about clinical trials?” with response options of Very knowledgeable, Somewhat knowledgeable, Not very knowledgeable, Not at all knowledgeable. Clinical trial knowledge was examined as a binary variable with categories Very/Somewhat knowledgeable and Not very/Not at all knowledgeable.

*Clinical trial risk* was measured with the question “In your opinion, how much risk, if any, is associated with participating in clinical trials?” with answer choices of A lot of risk, Some risk, No risk at all.

#### Effect Measure Modifier

Race and ethnicity were collected with the question: *“What is your race or ethnic background? (check all that apply)”* with response options of White; Hispanic, Latino, or Spanish origin; Black; Middle Eastern or North African; Native Hawaiian or Pacific Islander; Asian; American Indian, Native, First Nations, Indigenous Peoples of the Americas, or Alaska Native; Some other Race or Origin (please specify); Don’t Know/Not Sure; and Decline to state. For each racial category selected (besides Some other Race or Origin (please specify); Don’t Know/Not Sure; Decline to state), a follow-up question was asked: *“Which group(s) best represents your origin or ancestry?”* with answer choices specific to each racial group, Other (please specify), Don’t Know/Not Sure, and Decline to state. Language and birth country were used to recode missing ethnicities when applicable.

Using ethnic subgroup data, a mutually exclusive racial, ethnic, or regional identity variable with the categories Chinese, Korean, South Asian (including Asian Indian, Bangladeshi, Nepali, and Pakistani individuals), Haitian, North American Latiné (including Costa Rican, Cuban, Dominican, Guatemalan, Honduran, Mexican, Nicaraguan, Panamanian, Puerto Rican, and Salvadoran individuals), South American

Latiné (including Bolivian, Brazilian, Chilean, Colombian, Ecuadorian, Paraguayan, Peruvian, and Venezuelan individuals), and Middle Eastern or North African (now referred to as Southwest Asian or North African [SWANA]; including Afghan, Algerian, Egyptian, Iranian, Iraqi, Israeli, Jordanian, Lebanese, Moroccan, Palestinian, Syrian, and Yemeni individuals) was created. The split into North American Latiné and South American Latiné from the Latiné group was determined by geography, migration patterns, sample size, and a previous publication, which examined several North American Latiné groups (e.g., Central American, Dominican, Mexican) individually and South American Latiné collectively.^24^

All individuals identifying as more than one broader racial group (e.g., White, Asian) were excluded. While there are groups of White individuals who are often excluded from health research, including rural White communities, the present study aimed to focus on racial and ethnic research participation disparities. Therefore, White individuals were excluded from the present study. See the Appendix for further details on the racial, ethnic, or regional identity group variable.

#### Covariates

Age (continuous), birth sex (male, female), education level (binary variable with categories of High school graduate or less vs. More than high school [Some college/vocational/technical school or higher]), and nativity (binary variable with categories of Born in the U.S. vs. Not born in the U.S.) were examined as covariates.

#### Analyses

The Cancer CHRNA dataset included 2,636 participants; 2,042 identified as Chinese, Korean, South Asian, Haitian, North American Latiné, South American Latiné, or SWANA. Of the 2,042, 24 were excluded due to identifying as another racial or ethnic group/subgroup. Another 230 participants were excluded due to missing one or more of the variables described above. The analytic sample was 1,788. Frequencies and percentages of all categorical variables and means and standard deviations for all continuous variables were calculated overall and stratified by racial, ethnic, or regional group.

Chi-square tests for categorical variables and Kruskal-Wallis tests for continuous variables were used to compare characteristics across groups. Multinomial regression analysis was used to examine the associations between medical mistrust, clinical trial knowledge, and clinical trial risk and willingness to participate in health research (Yes (ref), No, Don’t know/Not sure). Effect measure modification of racial, ethnic, or regional identity on the association between each exposure (medical mistrust, clinical trial knowledge, clinical trial risk) and willingness to participate in health research was examined in three separate multinomial regression models. When the interaction term was significant (p < 0.10), models were stratified by racial, ethnic, or regional identity. Collinearity was assessed with correlation analysis; all exposures and covariates were included in the same model. All analyses were conducted in SAS Studio version 3.81. Interaction significance was assessed at *α=*0.1; main model significance was assessed at *α*=0.05.

## Results

The largest racial, ethnic, or regional group in the analytic sample was Chinese (30.0%) followed by South Asian (16.0%), SWANA (14.8%), Korean (13.6%), North American Latiné (12.1%), Haitian (8.3%), and South American Latiné (5.2%) (Table 1). Most of the sample was female (62.0%), had education beyond high school (57.1%), and were not born in the U.S. (79.0%). The mean age of the sample was 44.4 years. Close to half of participants (46.1%) were willing to participate in a future health research study, 27.6% were not willing to participate, and 26.3% were unsure of their willingness to participate. The mean medical mistrust score was 25.7. Most participants reported being Not very/Not at all knowledgeable about clinical trials (66.3%) and perceiving Some clinical trial risk (76.3%).

All variables differed significantly by racial, ethnic, or regional group (p < 0.0001) (Table 1). Mean age ranged from 37.4 years among South Asian participants to 49.4 years among Chinese participants. A larger proportion of females participated across all racial groups with the largest proportion of females among North American Latiné individuals (76.4%) and the smallest proportion among SWANA individuals (55.3%). South Asian participants were most likely to have had some education beyond high school (74.8%); Chinese participants were least likely (36.1%). Korean participants were most likely to have been born outside of the U.S. (86.4%); North American Latiné participants were least likely (59.3%). South Asian participants were most likely to be willing to participate in health research (58.7%); Chinese participants were least likely (35.8%). Medical mistrust scores ranged from 24.3 (least mistrust) among Chinese participants to 29.0 (most mistrust) among Haitian participants. South Asian and Haitian participants self-reported the most clinical trial knowledge: 45.1% and 45.0%, respectively, reported being Very/somewhat knowledgeable. Korean individuals were least likely to report being Very/somewhat knowledgeable (16.9%). Haitian participants were most likely to report A lot of risk associated with clinical trial participation (42.3%). Among the other groups, perception of A lot of risk ranged from 11.8% among South American Latiné individuals to 17.6% among North American Latiné individuals.

Evidence of effect measure modification was found for racial, ethnic, or regional identity on the relationship between medical mistrust (p < 0.0001), clinical trial knowledge (p = 0.08), and clinical trial risk (p = 0.03) and willingness to participate in health research (data not shown).

Table 2 presents the multinomial model outputs for the outcome of not being willing to participate in health research vs. being willing to participate in health research stratified by racial, ethnic, or regional identity. As medical mistrust increased, the odds of not being willing to participate vs. being willing to participate increased among Chinese (OR: 1.06, 95%CI: 1.00, 1.12) and South American Latiné (OR: 1.15, 95%CI: 1.01, 1.30) participants. Conversely, among Haitian participants, as medical mistrust increased, the odds of *not* being willing to participate vs. being willing to participate *decreased* (OR: 0.87, 95%CI: 0.81, 0.94).

Haitian participants who reported being Not very/Not at all knowledgeable compared to being Somewhat/Very knowledgeable about clinical trials had 2.77 (95%CI: 1.15, 6.65) times higher odds of *not* being willing to participate vs. being willing to participate. Conversely, Chinese participants who reported being Not very/Not at all knowledgeable about clinical trials compared to being Somewhat/Very knowledgeable had 0.55 (95%CI: 0.34, 0.88) times lower odds of *not* being willing to participate in health research vs. being willing to participate. South American Latiné individuals who reported perceiving Some risk compared to No risk associated with health research participation had 0.14 (95%CI: 0.02, 0.85) times *lower* odds of *not* being willing to participate in health research vs. being willing to participate.

Table 3 presents the multinomial model outputs for the outcome of *not knowing/being unsure* of willingness to participate in health research vs. being willing to participate in health research. As medical mistrust increased, the odds of not knowing/being unsure of willingness to participate vs. being willing to participate increased among Korean (OR: 1.13, 95%CI: 1.05, 1.22), South Asian (OR: 1.07, 95%CI: 1.01, 1.12), and North American Latiné (OR: 1.18, 95%CI: 1.09, 1.27) participants. Conversely, as medical mistrust increased, the odds of not knowing/being unsure of willingness to participate vs. being willing to participate decreased among Haitian (OR: 0.91, 95%CI: 0.84, 0.99) and SWANA (OR: 0.91, 95%CI: 0.86, 0.97) participants. South Asian and SWANA participants who reported being Not very/Not at all knowledgeable of clinical trials compared to being Somewhat/Very knowledgeable of clinical trials had higher odds of not knowing/being unsure of willingness to participate vs. being willing to participate (OR: 2.09, 95%CI: 1.07, 4.07; OR: 2.71, 95%CI: 1.21, 6.03, respectively).

## Discussion

In this study across seven NYC racial, ethnic, or regional groups, nearly half of the analytic sample expressed willingness to participate in future health research studies, with variation across groups. This level of willingness aligns with the levels among the general U.S. population,^25^ challenging assumptions around minoritized populations not trusting research. Levels of medical mistrust, clinical trial knowledge, and perceived clinical trial risk and their association with willingness to participate varied by group, indicating heterogeneity in factors influencing research participation across diverse groups in NYC.

Although we examined willingness to not participate, interpreting our findings within the context of existing literature is more easily understood when framed as less willingness to participate. We found that increased medical mistrust was associated with *less willingness* to participate in future health studies among Chinese and South American Latiné participants; among Haitian participants, increased mistrust was associated with *increased* willingness to participate. Among Korean, South Asian, and North American Latiné participants, more mistrust was associated with higher odds of *not knowing* their willingness to participate (vs. being willing). Conversely, more mistrust and *lower* odds of not knowing their willingness to participate were associated among Haitian and SWANA participants.

Chinese participants reporting less clinical trial knowledge were more likely to be willing to participate, while Haitian participants reporting less clinical trial knowledge were less likely to be willing. South Asian and SWANA participants reporting less clinical trial knowledge had higher odds of not knowing their willingness. South American Latiné participants reporting Some clinical trial risk (vs. No risk at all) were more likely to be willing to participate in future health research.

To our knowledge, this study is the first to examine the associations of medical mistrust, clinical trial knowledge, and perceived clinical trial risk with research participation willingness among the included NYC groups. Our findings indicate the need for tailored research recruitment interventions–including collaboration with CBOs and community health workers (CHWs)–and are important for guiding efforts to increase research participation across diverse groups.

### Willingness to participate

Our findings align previous studies indicating differences in willingness to participate across diverse groups,^26, 27^ including Liu et al.’s 2019 study among 17,339 Asian, Black, Latiné, Caucasian, and “other minority” U.S. residents.^27^ While our findings on differences in participation willingness across ethnic groups align, Lui et al. consistently found higher rates of participation willingness among their racial and ethnic groups compared the subgroups examined in our study (i.e., Liu’s Asian Americans had higher rates than Chinese and Korean participants of this study, Black Americans had a higher rate than Haitian participants, and Latiné participants had higher rates than North and South American Latiné participants).^27^

Our lower rates may stem from nearly 80% of our sample having immigrated to the U.S., with just over half speaking English well or very well. Immigrants face unique challenges to research participation, including limited access to insurance and health information, and concerns about legal consequences.^28^ Moreover, underrepresentation of immigrant populations in health and research professions may prevent their use of lived experiences to inform inclusive recruitment and retention practices.^28^

### Medical mistrust

Findings on the association between medical mistrust and willingness to participate varied by group. Findings among Chinese, Korean, South Asian, and North and South American Latiné participants aligned with previous findings among Chinese, Asian, and Latiné individuals in the U.S., which indicate mistrust is inversely associated with participation willingness.^4^ However, increased mistrust was positively associated with participation willingness among Haitian and SWANA participants, a finding both counterintuitive and missing from the literature among Black and Asian Americans. A CHW who collected data for the Cancer CHRNA indicated that this finding may be partially explained by health literacy, which was not captured in the Cancer CHRNA survey. Health literacy could be linked to both more awareness of historical mistreatment of minoritized groups, leading to more medical mistrust, and more awareness of the benefits of clinical research and current protections for research participants, leading to more willingness to participate.

Existing literature among racial and ethnic groups on the association between health literacy and a) medical mistrust and b) willingness to participate is varied.^29^ Tsai et al.’s 2018 study among Taiwanese individuals^30^ indicates a positive relationship between trust and health literacy, while White et al.’s 2014 study among Latiné individuals indicates a negative relationship.^31^ A 2020 review of barriers to health literacy among African Americans found medical mistrust to be a barrier to health literacy,^32^ yet, a 2015 study among Haitian American immigrants found no significant difference in health literacy between those with little to no trust in doctors compared to those with some or a lot of trust.^33^ Considering the variation in findings and lack of research among Haitian and SWANA U.S. residents, additional qualitative research—conducted with input from CBOs and CHWs—into the relationships between health literacy, medical mistrust, and willingness to participate among these groups is needed.

### Clinical trial knowledge

Among Haitian, South Asian, and SWANA participants, less clinical trial knowledge was associated with less or unknown willingness to participate in health research, aligning with previous findings among African American and Asian American individuals.^4^ However, among Chinese participants, *less* knowledge was associated with *more* willingness to participate. Several factors could be responsible, including trust in healthcare providers mediating the relationship between less knowledge and participation willingness. A 2014 study among 3,159 Chicago-area Chinese adults aged ≥ 60 years found that participants had high levels of trust in physicians, and that being female, being born in China, and having lower education was associated with this increased physician trust.^34^ Given that these participants’ characteristics reflect the majority of Chinese participants in our study, and that lower education and less clinical trial knowledge are correlated, it is possible that elevated healthcare provider trust underpins the observed associations. Findings from a focus group study among 103 Asian Americans in Philadelphia, most of whom were of Chinese descent, found doctor recommendations to be a motivator for clinical trial participation and lack of trust in healthcare providers to be a barrier.^35^ These findings, though sparse, indicate the potential for mediation, however, should be followed up with further studies examining these relationships among Chinese individuals.

### Clinical trial risk

Among SWANA participants, Some perceived clinical trial risk—compared to No perceived risk—was associated with more willingness to participate in health research. This counterintuitive finding may be due to > 80% of SWANA participants indicating Some perceived clinical trial risk, resulting in small sample sizes for the other risk categories. This trend was consistent across racial and ethnic groups and may contribute to the lack of findings overall between perceived risk and participation willingness.

### Strengths and Limitations

This study has several major strengths, including collecting detailed racial-, ethnic-, and national-level data—allowing for disaggregated analyses across ethnic, national, and regional groups—along with extensive collaboration with a large, multilingual CBO and CHW team to recruit for and administer the survey, which created novel opportunities to engage limited English proficiency individuals in research. This resulted in historically underrepresented groups being part of this analysis. An important limitation of the study is the study population being a convenience sample, limiting generalizability. Another limitation is the collection of perceived clinical trial risk as a three-category variable which likely led to most participants selecting the middle category of “some risk” and the subsequent lack of findings between risk and willingness to participate.

## Conclusions

This study is the first, to our knowledge, to examine the associations of medical mistrust, trial knowledge, and perceived trial risk with willingness to participate in health research among Chinese, Korean, South Asian, Haitian, North American Latiné, South American Latiné, and SWANA individuals in the U.S. The varied findings in presence, direction, and strength of associations of medical mistrust, clinical trial knowledge, and perceived clinical trial risk with willingness to participate in future health research across groups, along with the lack of U.S. research participation among diverse individuals, indicate a need for more racially and ethnically disaggregated research into factors impacting participation along with tailored interventions to improve participation among these groups. It is imperative to work with CBOs and CHWs from the communities that studies aim to reach to ensure culturally appropriate and linguistically tailored recruitment. This will demonstrate representation of target participant groups within the medical research system, building participant trust with the study team and the health research process overall.

## Supplementary Files

This is a list of supplementary files associated with this preprint. Click to download.

- Appendix.docx

## Figures and Tables

**Table 1:** Descriptive statistics by racial or ethnic identity

Variable	Total Sample N (%)	Chinese N (%)	Korean N (%)	South Asian* N (%)	Haitian N (%)	North American Latiné** N (%)	South American Latiné*** N (%)	SWANA**** N(%)	P-value
Total, n (%)	1,788	537 (30.0%)	243 (13.6%)	286 (16.0%)	149 (8.3%)	216 (12.1%)	93 (5.2%)	264 (14.8%)	
*Willingness to participate in health research*									**< 0.0001**
Yes	824 (46.1%)	192 (35.8%)	116 (47.7%)	168 (58.7%)	57 (38.3%)	114 (52.8%)	49 (52.7%)	128 (48.5%)	
No	493 (27.6%)	176 (32.8%)	75 (30.9%)	60 (21.0%)	59 (39.6%)	39 (18.1%)	17 (18.3%)	67 (25.4%)	
Don’t know/Not sure	471 (26.3%)	169 (31.5%)	52 (21.4%)	58 (20.3%)	33 (22.1%)	63 (29.2%)	27 (29.0%)	69 (26.1%)	
*Medical mistrust, mean (SD)*	25.7 (5.4)	24.3 (4.0)	25.3 (5.3)	24.8 (6.2)	29.0 (6.4)	26.8 (5.0	27.7 (5.5)	24.9 (5.5)	**< 0.0001**
*Clinical trial knowledge*									**< 0.0001**
Very/Somewhat knowledgeable	602 (33.7%)	147 (27.4%)	41 (16.9%)	129 (45.1%)	67 (45.0%)	93 (43.1%)	40 (43.0%)	85 (32.2%)	
Not very/Not at all knowledgeable	1186 (66.3%)	390 (72.6%)	202 (83.1%)	157 (54.9%)	82 (55.0%)	123 (56.9%)	53 (57.0%)	179 (67.8%)	
*Clinical trial risk*									**< 0.0001**
A lot of risk	307 (17.2%)	85 (15.8%)	35 (14.4%)	43 (15.0%)	63 (42.3%)	38 (17.6%)	11 (11.8%)	32 (12.1%)	
Some risk	1364 (76.3%)	439 (81.8%)	194 (79.8%)	206 (72.0%)	78 (52.4%)	162 (75.0%)	72 (77.4%)	213 (80.7%)	
No risk at all	117 (6.5%)	13 (2.4%)	14 (5.8%)	37 (13.0%)	8 (5.4%)	16 (7.4%)	10 (10.8%)	19 (7.2%)	
*Age, mean (SD)*	44.4 (17.4)	49.4 (18.0)	45.3 (17.6)	37.4 (14.0)	37.7 (14.9)	41.5 (15.0)	43.4 (15.2)	47.1 (18.8)	**< 0.0001**
*Sex assigned at birth*									**< 0.0001**
Male	679 (38.0%)	201 (37.4%)	106 (43.6%)	124 (43.4%)	55 (36.9%)	51 (23.6%)	24 (25.8%)	118 (44.7%)	
Female	1109 (62.0%)	336 (62.6%)	137 (56.4%)	162 (56.6%)	94 (63.1%)	165 (76.4%)	69 (74.2%)	146 (55.3%)	
*Education level*									**< 0.0001**
High school graduate or less	768 (43.0%)	343 (63.9%)	71 (29.2%)	72 (25.2%)	38 (25.5%)	117 (54.2%)	44 (47.3%)	83 (31.4%)	
More than high school	1,020 (57.1%)	194 (36.1%)	172 (70.8%)	214 (74.8%)	111 (74.5%)	99 (45.8%)	49 (52.7%)	181 (68.6%)	
*Nativity*									**< 0.0001**
Born in the United States	375 (21.0%)	82 (15.3%)	33 (13.6%)	71 (24.8%)	32 (21.5%)	88 (40.7%)	20 (21.5%)	49 (18.6%)	
Not born in the United States	1413 (79.0%)	455 (84.7%)	210 (86.4%)	215 (75.2%)	117 (78.5%)	128 (59.3%)	73 (78.5%)	215 (81.4%)	

* Includes Asian Indian, Bangladeshi, Nepali, and Pakistani individuals

** Includes Costa Rican, Cuban, Dominican, Guatemalan, Honduran, Mexican, Nicaraguan, Panamanian, Puerto Rican, and Salvadoran individuals

*** Includes Bolivian, Brazilian, Chilean, Colombian, Ecuadorian, Paraguayan, Peruvian, and Venezuelan individuals

**** Southwest Asian and North African; includes Afghan, Algerian, Egyptian, Iranian, Iraqi, Israeli, Jordanian, Lebanese, Moroccan, Palestinian, Syrian, and Yemeni individuals

**Table 2:** Multinomial regression models evaluating the odds of not being willing to participate in health research among Chinese, Korean, South Asian, Haitian, North American Latiné, South American Latiné, and SWANA study participants

Variable	Chinese N = 537		Korean N = 243		South Asian* N = 286		Haitian N = 149		North American Latiné** N = 216		South American Latiné*** N = 93		SWANA**** N = 264	
	OR [95% Cl]	P-value	OR [95% Cl]	P-value	OR [95% Cl]	P-value	OR [95% Cl]	P-value	OR [95% Cl]	P-value	OR [95% Cl]	P-value	OR [95% Cl]	P-value
*Medical mistrust. (continuous)*	**1.06 [1.00, 1.12]**	**< 0.05**	1.05 [0.98, 1.12]	0.16	0.99 [0.94, 1.04]	0.55	**0.87 [0.81, 0.94]**	**< 0.001**	1.08 [1.00, 1.17]	0.06	**1.15 [1.01, 1.30]**	**0.03**	0.95 [0.89, 1.00]	0.07
*Clinical trial knowledge*														
Very/Somewhat knowledgeable (ref)	--	**--**	**--**	**--**	**--**	**--**	--	--			--	--	--	--
Not very/Not at all knowledgeable	**0.55 [0.34, 0.88]**	**0.01**	1.27 [0.50, 3.24]	0.61	1.11 [0.60, 2.06]	0.74	**2.77 [1.15, 6.65]**	**0.02**	1.59 [0.74, 3.44]	0.24	0.45 [0.11, 1.83]	0.26	0.61 [0.32, 1.17]	0.14
*Clinical trial risk*														
No risk at all (ref)	--	**--**	**--**	**--**	**--**	**--**	--	--			--	--	--	--
Some risk	2.31 [0.55, 9.75]	0.25	0.36 [0.09, 1.40]	0.14	2.00 [0.68, 5.82]	0.21	3.85 [0.67, 22.02]	0.13	1.00 [0.24, 4.08]	0.99	**0.14 [0.02, 0.85]**	**0.03**	0.86 [0.23, 3.16]	0.82
A lot of risk	2.22 [0.49, 10.14]	0.30	1.44 [0.29, 7.23]	0.66	2.85 [0.81, 10.07]	0.10	2.31 [0.39, 13.85]	0.36	0.72 [0.13, 3.82]	0.70	0.16 [0.01, 2.18]	0.17	1.53 [0.34, 6.84]	0.58
*Age (continuous)*	1.00 [0.98, 1.01]	0.47	**1.07 [1.04, 1.10]**	**< 0.0001**	1.01 [0.98, 1.03]	0.54	1.02 [0.99, 1.05]	0.23	1.01 [0.98, 1.03]	0.70	0.96 [0.91, 1.01]	0.14	1.01 [0.98, 1.03]	0.59
*Sex assigned at birth*														
Male (ref)	--	**--**	**--**	**--**	**--**	**--**	--	--			--	--	--	--
Female	0.95 [0.62, 1.47]	0.83	0.68 [0.34, 1.36]	0.28	0.93 [0.50, 1.72]	0.81	0.50 [0.21, 1.22]	0.13	0.71 [0.29, 1.78]	0.47	**0.12 [0.02, 0.54]**	**< 0.01**	0.72 [0.39, 1.33]	0.29
*Education level*														
More than high school (ref)	--	**--**	**--**	**--**	**--**	**--**	--	--			--	--	--	--
High school graduate or less	**2.35 [1.44, 3.85]**	**< 0.001**	0.99 [0.46, 2.12]	0.98	1.80 [0.91, 3.55]	0.09	**0.35 [0.13, 0.97]**	**0.04**	1.00 [0.45, 2.21]	0.99	0.87 [0.22, 3.47]	0.84	0.93 [0.45, 1.93]	0.84
*Nativity*														
Born in the United States (ref)	--	**--**	**--**	**--**	**--**	**--**	--	--			--	--	--	--
Not born in the United States	0.99 [0.51, 1.93]	0.97	0.57 [0.19, 1.67]	0.30	1.70 [0.73, 3.97]	0.22	1.36 [0.49, 3.80]	0.56	0.61 [0.28, 1.33]	0.21	6.25 [0.91, 42.93]	0.06	**0.38 [0.15, 0.99]**	**0.05**

* Includes Asian Indian, Bangladeshi, Nepali, and Pakistani individuals

** Includes Costa Rican, Cuban, Dominican, Guatemalan, Honduran, Mexican, Nicaraguan, Panamanian, Puerto Rican, and Salvadoran individuals

*** Includes Bolivian, Brazilian, Chilean, Colombian, Ecuadorian, Paraguayan, Peruvian, and Venezuelan individuals

**** Southwest Asian and North African; includes Afghan, Algerian, Egyptian, Iranian, Iraqi, Israeli, Jordanian, Lebanese, Moroccan, Palestinian, Syrian, and Yemeni individuals

OR, odds ratio; CI, confidence interval

**Table 3:** Multinomial regression models evaluating the odds of not knowing or not being sure of willingness to participate in health research among Chinese, Korean, South Asian, Black, and Latiné study participants

Variable	Chinese N = 537		Korean N = 243		South Asian* N=286		Haitian N = 149		North American Latiné** N = 216		South American Latiné*** N = 93		SWANA**** N = 264	
	OR [95% Cl]	P-value	OR [95% Cl]	P-value	OR [95% CO	P-value	OR [95% CO	P-value	OR [95% Cl]	P-value	OR [95% Cl]	P-value	OR [95% Cl]	P-value
*Medical mistrust (continuous)*	1.05 [0.99, 1.11]	0.09	**1.13 [1.05, 1.22]**	**< 0.01**	**1.07 [1.01, 1.12]**	**0.02**	**0.91 [0.84, 0.99]**	**0.03**	**1.18 [1.09, 1.27]**	**< 0.0001**	0.98 [0.89, 1.08]	0.66	**0.91 [0.86, 0.97]**	**< 0.01**
*Clinical trial knowledge*														
Very/Somewhat knowledgeable (ref)	--	**--**	**--**	**--**	**--**	**--**	--	--			**--**	**--**	--	--
Not very/Not at all knowledgeable	1.11 [0.66, 1.85]	0.69	1.06 [0.40, 2.83]	0.91	**2.09 [1.07, 4.07]**	**0.03**	2.68 [0.98, 7.35]	0.06	1.83 [0.91. 3.68]	0.09	2.34 [0.78, 7.01]	0.13	**2.71 [1.21, 6.03]**	**0.02**
*Clinical trial risk*														
No risk at all (ref)	--	**--**	**--**	**--**	**--**	**--**	--	--			--	--	--	--
Some risk A lot of risk	1.41 [0.38, 5.20]	0.61	2.62 [0.27, 25.67]	0.41	0.80 [0.32, 1.98]	0.63	7.22 [0.64, 81.22]	0.11	0.97 [0.26, 3.62]	0.96	3.49 [0.36, 34.13]	0.28	0.39 [0.13, 1.20]	0.10
	1.59 [0.40, 6.37]	0.51	5.33 [0.44, 64.24]	0.19	0.89 [0.28, 2.81]	0.84	5.48 [0.48, 63.13]	0.17	1.04 [0.24, 4.49]	0.96	1.07 [0.07, 16.57]	0.96	0.34 [0.08, 1.55]	0.16
*Age (continuous)*	1.00 [0.99, 1.02]	0.90	**1.06 [1.03, 1.09]**	**< 0.0001**	1.01 [0.99, 1.04]	0.42	**1.05 [1.01, 1.09]**	**< 0.01**	1.00 [0.98, 1.02]	0.95	0.99 [0.96, 1.03]	0.66	0.99 [0.97, 1.01]	0.17
*Sex assigned at birth*														
Male (ref)	--	**--**	**--**	**--**	**--**	**--**	--	--			--	--	--	--
Female	1.13 [0.73, 1.75]	0.60	2.02 [0.91, 4.50]	0.08	1.20 [0.63, 2.28]	0.59	**0.35 [0.13, 0.96]**	**0.04**	**0.39 [0.18, 0.85]**	**0.02**	1.74 [0.47, 6.39]	0.40	0.68 [0.36, 1.29]	0.24
*Education level*														
More than high school (ref)	--	**--**	**--**	**--**	**--**	**--**	--	--			--	--	--	--
High school graduate or less	**1.76 [1.08, 2.87]**	**0.02**	0.64 [0.26, 1.56]	0.32	1.13 [0.54, 2.37]	0.74	0.87 [0.30, 2.51]	0.80	1.15 [0.56, 2.36]	0.71	1.43 [0.48, 4.26]	0.52	0.64 [0.30, 1.38]	0.27
*Nativity*														
Born in the United States (ref)	--	**--**	**--**	**--**	**--**	**--**	--	--			--	--	--	--
Not born in the United States	0.64 [0.34, 1.22]	0.17	1.54 [0.42, 5.68]	0.52	1.19 [0.53, 2.72]	0.67	1.99 [0.51, 7.80]	0.33	0.98 [0.48, 2.01]	0.95	1.78 [0.40, 8.00]	0.45	1.44 [0.51, 4.09]	0.49

* Includes Asian Indian, Bangladeshi, Nepali, and Pakistani individuals

** Includes Costa Rican, Cuban, Dominican, Guatemalan, Honduran, Mexican, Nicaraguan, Panamanian, Puerto Rican, and Salvadoran individuals

*** Includes Bolivian, Brazilian, Chilean, Colombian, Ecuadorian, Paraguayan, Peruvian, and Venezuelan individuals

**** Southwest Asian and North African; includes Afghan, Algerian, Egyptian, Iranian, Iraqi, Israeli, Jordanian, Lebanese, Moroccan, Palestinian, Syrian, and Yemeni individuals

OR, odds ratio; CI, confidence interval

## Data Availability

Data generated in this study are not publicly available due to information that could compromise patient privacy/consent but are available upon reasonable request from the corresponding author.
